# SARS-CoV-2 variants of concern dominate in Lahore, Pakistan in April 2021

**DOI:** 10.1099/mgen.0.000693

**Published:** 2021-11-30

**Authors:** Muhammad Bilal Sarwar, Muhammad Yasir, Nabil-Fareed Alikhan, Nadeem Afzal, Leonardo de Oliveira Martins, Thanh Le Viet, Alexander J. Trotter, Sophie J. Prosolek, Gemma L. Kay, Ebenezer Foster-Nyarko, Steven Rudder, David J. Baker, Sidra Tul Muntaha, Muhammad Roman, Mark A. Webber, Almina Shafiq, Bilquis Shabbir, Javed Akram, Andrew J. Page, Shah Jahan

**Affiliations:** ^1^​ Department of Immunology, University of Health Sciences, Lahore, Pakistan; ^2^​ Quadram Institute Bioscience, Norwich Research Park, Norwich, Norfolk, UK; ^3^​ Central Diagnostic Facility, Mayo Hospital, Lahore, Pakistan; ^4^​ University of East Anglia, Norwich, Norfolk, UK; ^5^​ Department of Medicine, East Medical Ward, King Edward Medical University Mayo Hospital, Lahore, Pakistan

**Keywords:** ARTIC, genomic epidemiology, genome, NGS, sequencing, SARS-CoV-2

## Abstract

The SARS-CoV-2 pandemic continues to expand globally, with case numbers rising in many areas of the world, including the Indian sub-continent. Pakistan has one of the world’s largest populations, of over 200 million people and is experiencing a severe third wave of infections caused by SARS-CoV-2 that began in March 2021. In Pakistan, during the third wave until now only 12 SARS-CoV-2 genomes have been collected and among these nine are from Islamabad. This highlights the need for more genome sequencing to allow surveillance of variants in circulation. In fact, more genomes are available among travellers with a travel history from Pakistan, than from within the country itself. We thus aimed to provide a snapshot assessment of circulating lineages in Lahore and surrounding areas with a combined population of 11.1 million. Within a week of April 2021, 102 samples were sequenced. The samples were randomly collected from two hospitals with a diagnostic PCR cutoff value of less than 25 cycles. Analysis of the lineages shows that the Alpha variant of concern (first identified in the UK) dominates, accounting for 97.9 % (97/99) of cases, with the Beta variant of concern (first identified in South Africa) accounting for 2.0 % (2/99) of cases. No other lineages were observed. In depth analysis of the Alpha lineages indicated multiple separate introductions and subsequent establishment within the region. Eight samples were identical to genomes observed in Europe (seven UK, one Switzerland), indicating recent transmission. Genomes of other samples show evidence that these have evolved, indicating sustained transmission over a period of time either within Pakistan or other countries with low-density genome sequencing. Vaccines remain effective against Alpha, however, the low level of Beta against which some vaccines are less effective demonstrates the requirement for continued prospective genomic surveillance.

## Data Summary

Consensus genomes are available from GISAID (Shu and McCauley, 2017) subject to minimum quality control criteria, with individual sample accession numbers in Table S1, available in the online version of this article). Raw reads and consensus genomes are available from European Nucleotide Archive (ENA) in Bioproject PRJEB45462. Individual sample accession numbers for each database are listed in Table S1. Anonymized metadata was available to all authors and is deposited along with the genomic data in GISAID and the ENA and is provided in Table S1. Supplementary Material can be found in Figshare: https://doi.org/10.6084/m9.figshare.16553199.v1 [1].

**Impact Statement**

Genome sequencing of SARS-CoV-2 helps public health scientists to understand the dynamics of the pandemic in different parts of the world. Low- and middle-income countries have low levels of genomic surveillance, depriving them of effective data sources to manage their pandemic response and set public health policy. We provide a snapshot of samples taken over 1 week from Lahore, Pakistan and find that all of the samples sequenced were variants of concern, which have either increased transmissibility or reduced efficacy against vaccines. The 102 samples sequenced represented a fold increase in the number of genomes available for Pakistan (at the time of sequencing).

## Introduction

The COVID-19 pandemic has spread rapidly throughout the world and continues to expand in many regions. It began with an unknown case of pneumonia in the city of Wuhan, PR China [2]. The causative pathogen has since been named ‘severe acute respiratory syndrome-related coronavirus 2 (SARS-CoV-2)’. As of May 2021, there have been over 167 million reported cases and 3.4 million fatalities [3]. Genomic surveillance has assisted the pandemic response providing information for outbreak investigations and detecting possible epitope changes that would allow the virus to escape vaccines. Multiple classification systems have been developed to quickly communicate SARS-CoV-2 variants circulating in a community [4–6]. From these definitions, certain lineages have been designated as variants of concern (VOCs), which are defined as such due to indications of increased transmission patterns and/or possible resistance to vaccine and/or other treatments [7]. The World Health Organisation (WHO) has introduced a new nomenclature of these VOCs and variants of Interest (VOIs) based on Greek alphabets.

B.1.1.7 (Alpha) and B.1.351 (Beta) are two VOCs that have circulated globally. The SARS-CoV-2 lineage B.1.1.7, designated variant of concern 202012/01 (VOC) by Public Health England, was first identified in the UK in late Summer to early Autumn 2020 [7]. B.1.351 is another VOC identified in South Africa and defined by eight mutations in the spike protein including (K417N, E484K and N501Y) [8].

Pakistan is currently experiencing a severe third wave of infections caused by SARS-CoV-2, which began in March 2021. Vaccination rates nationally are under 2 % [9], leaving large segments of the community at risk of serious illness from COVID-19. Currently very few SARS-CoV-2 genomes collected in Pakistan are available, with just 12 covering the third wave, nine of which are from one city, Islamabad. This highlights the need for more genome sequencing from Pakistan, particularly given the current situation and the very high population in order to allow surveillance of variants in circulation. Currently, more genomes are available in the GISAID database for travellers with a travel history from Pakistan, than from within the country itself [10].

We have amplicon sequenced 102 samples, randomly chosen from a 1 week period in April 2021 from Lahore and surrounding areas, to get a snapshot assessment of the circulating lineage in the region. This has identified the Alpha variant of concern as the primary lineage circulating, found in 97.9 % of cases, with clear signals of repeated overseas introductions into the region.

## Methods

### Genome sequencing and analysis

RNA was extracted using Viral RNA Extraction kit, FavorPrep (Cat. No. FAVNK 001–1) and and detection was carried out on IQ5five real time PCR using GenomeCoV19 Detection Kit by abm (cat: 628) in facility of resource lab for research in biomedical sciences and COVID-19 research lab of University of Health Sciences, Lahore, Pakistan.

Positive samples with a CT <25 were randomly selected for genome sequencing. Viral RNA was converted in cDNA (SuperScript III) and was amplified using the ARTIC protocol v3 (LoCost) [11] with sequencing libraries prepared using CoronaHiT [12]. We carried out genome sequencing using the Illumina NextSeq 500 platform.

The raw reads were demultiplexed using bcl2fastq (v2.20). The reads were used to generate a consensus sequence using the ARTIC bioinformatic pipeline [13]. Briefly, the reads had adapters trimmed with TrimGalore [14] and were aligned to the WuhanHu-1 reference genome (accession MN908947.3) using BWA-MEM (v0.7.17) [15]; the ARTIC amplicons were trimmed and a consensus built using iVAR (v.1.2.3) [16].

PANGO lineages assigned using Pangolin v2.4.2 and PangoLEARN model dated 12 May 2021 [6, 17].

### Phylogenetic analysis

For the phylogenetic analysis, all sequences from GISAID (available on 14 May 2021) where Pakistan is the country of exposure were downloaded and added to the sequences from the current study. From these 268 samples, 131 were sequenced outside Pakistan, with 110 from the Airport Quarantine Station in Japan (travellers from Pakistan sampled at arrival) [18]. All remaining sequences from GISAID were then compared to this data set where we kept the closest ones – for each Pakistan sequence, the four closest from non-Pakistan were kept for subsequent analysis to provide context. The alignment and neighbour search were done with uvaia [19], and problematic (homoplasic or difficult to sequence) sites were masked from the alignment [20]. For the B.1.1.7 (Alpha) and B.1.351 (Beta) dated phylogenetic inference, we further enriched each alignment with more distant neighbours, which maximized the phylogenetic diversity [21], based on the neighbour-joining tree [22] of all sequences close to each Pakistan sequence, for each lineage. Sequences with more than 10 % N, or with incomplete date information were excluded from analysis. Clusters distant and unrelated to Pakistan sequences were reduced or removed by visual inspection of maximum-likelihood trees.

From the 102 UHS-PAK sequences, 90 samples were included in the phylogenetic analysis: 88 from Alpha and 2 samples from Beta; the final alignments have 723 and 107 sequences, respectively. The maximum-likelihood trees were inferred with IQTREE2 v2.1.2 [23] with 1000 ultrafast bootstrap replicates [24]. The divergence times were estimated by marginalization under a relaxed clock using TreeTime [25] and assuming a coalescent prior on three lengths with a constant effective population size. Polytomies were resolved by treetime when possible by using the time information. Ancestral state reconstruction of the country of exposure was done under parsimony over the timed tree with castor [26] and trees were plotted with ggtree [27], both for R. By weighting reconstructed states (countries of exposure) at the internal nodes by the number of most parsimonious scenarios, castor gives the probability of each node belonging to Pakistan or abroad. The number of introductions into Pakistan was estimated by counting branches where the reconstructed probability of being exposed in Pakistan increased to one. Equivalently, the exportations out of Pakistan were given by branches with a parental node most probably from Pakistan (prob >0.5) and a child node certainly not from Pakistan (prob=0). We assume that the date of the event is given by the parental node, to discount for ‘importation’ and ‘detection’ lags [28], and thus may be biassed since the true transition happened somewhere along the branch (i.e. more recent than our estimate). For this analysis we excluded the 21 Pakistani samples sequenced outside Pakistan, which might have been circulating abroad (i.e. except those from the Japanese Airport Quarantine Station). Notice that if the parsimony assumption fails, e.g. if we expect frequent international transmission events hidden along nodes in the tree, then this should be modelled under a probabilistic (likelihood or Bayesian) approach. Since we included the closest sequences from abroad, the reconstruction should not change by adding more available data. However, due to unequal sampling and sequencing, the number of transitions is an underestimate and their dates are subject to selection bias (e.g. previous introductions were not sequenced due to regional differences or severity of infection).

The distances between all leaf pairs along the maximum-likelihood tree were calculated with the package ape for R. These so-called patristic (or cophenetic) distances describe the path length along all branches separating two samples, and by taking the phylogeny into account are a better descriptor of the evolutionary differences between the sequences. By multiplying the patristic distances by genome length we obtain the expected number of SNP differences between samples, since branch lengths in a maximum-likelihood tree are given in ‘expected substitutions per site’.

## Results and discussion

In this study, from 6 April 2021 to 11 April 2021, 102 SARS-CoV-2 samples in viral transport medium (VTM) were randomly selected from SARS-CoV-2 diagnostic positives by the Mayo Hospital and Sheikh Zayed Hospital in Lahore, Pakistan. All samples had a Ct <25. These public hospitals serve a predominantly middle-income population. The cases were between 21 and 91 years of age with a broad distribution of ages, with 74 % (*n*=74) male, 26 % (*n*=26) female and two unknown. Four samples were from cases who died (all aged over 60), while all others recovered. Cases had no history of international travel, with 77 cases explicitly reporting that they and their household did not recently travel overseas (Table S1).

Viral RNA was amplified using the ARTIC protocol [11] with sequencing libraries prepared using CoronaHiT [12]. Resulting sequenced reads were used to generate consensus sequences with the ARTIC bioinformatics protocol (see Methods).

Analysis of the PANGO lineages shows that B.1.1.7 (Alpha) (first identified in the UK) dominates, accounting for 97.9 % (97/99) of cases, with B.1.351 (Beta) (first identified in South Africa) accounting for 2.0 % (2/99) of cases. Three further samples failed QC, yielding genomes less than 50 % coverage of SARS-CoV-2, and lineages could not be confidently called. No other lineages were observed.

SARS-CoV-2 was first identified in Pakistan on 26 February 2020 [29]. The first Alpha genome was submitted to GISAID on 25 December 2020. There were previously 268 SARS-CoV-2 genomes available on GISAID where Pakistan was listed as the country of exposure with 90 assigned as Alpha or Beta (Tables 1 and S1). Most of these samples were associated with international travel from the country and were collected outside of Pakistan (Table S2). A list of submitting authors of these data can be found in Table S3. We combined all Alpha and Beta public data with the genomes presented in this study to provide a snapshot of Alpha and Beta dissemination (Table S4).

**Table 1.** PANGO lineages of GISAID (as of 2020-05-14) data where Pakistan is the country of exposure, with collection dates of first and most recent samples

Lineage	Count	Oldest sample	Most recent sample	Lineage	Count	Oldest sample	Most recent sample
B.1.1.7 (Alpha)	87	25 December 2020	27 April 2021	B.1.36.22	1		30 November
B.1.351 (Beta)	3	9 April 2021	26 April 2021	C.23	5	11 May 2020	26 November 2020
B.1.617.1	1		26 April 2021	B.1	30	3 May 2020	24 November 2020
C.36	1		26 March 2021	B.1.36.8	1		24 November 2020
B.1.525	1		19 February 2021	B.1.36.24	3	2020-10-31	13 November 2020
B.1.1	10	11 May 2020	14 February 2021	B.1.459	1		10 October 2020
B.1.36	35	2 June 2020	9 February 2021	B.1.562	1		10 October 2020
B.1.36.31	17	10 October 2020	23 January 2021	B	3	12 March 2020-	2 July 2020
B.1.36.34	5	9 September 2020	11 January 2021	B.1.260	1		2 July 2020
B.1.1.214	1		6 January 2021	C.11	2	3 June 2020	18 June 2020
B.1.523	1		5 January 2021	B.6	12	16 March 2020	14 June 2020
B.1.1.1	18	3 June 2020	14 December 2020	A	3	2 June 2020	3 June 2020
B.1.471	24	20 May 2020	14 December 2020	B.1.1.370	1		28 May 2020

**Fig. 1.**
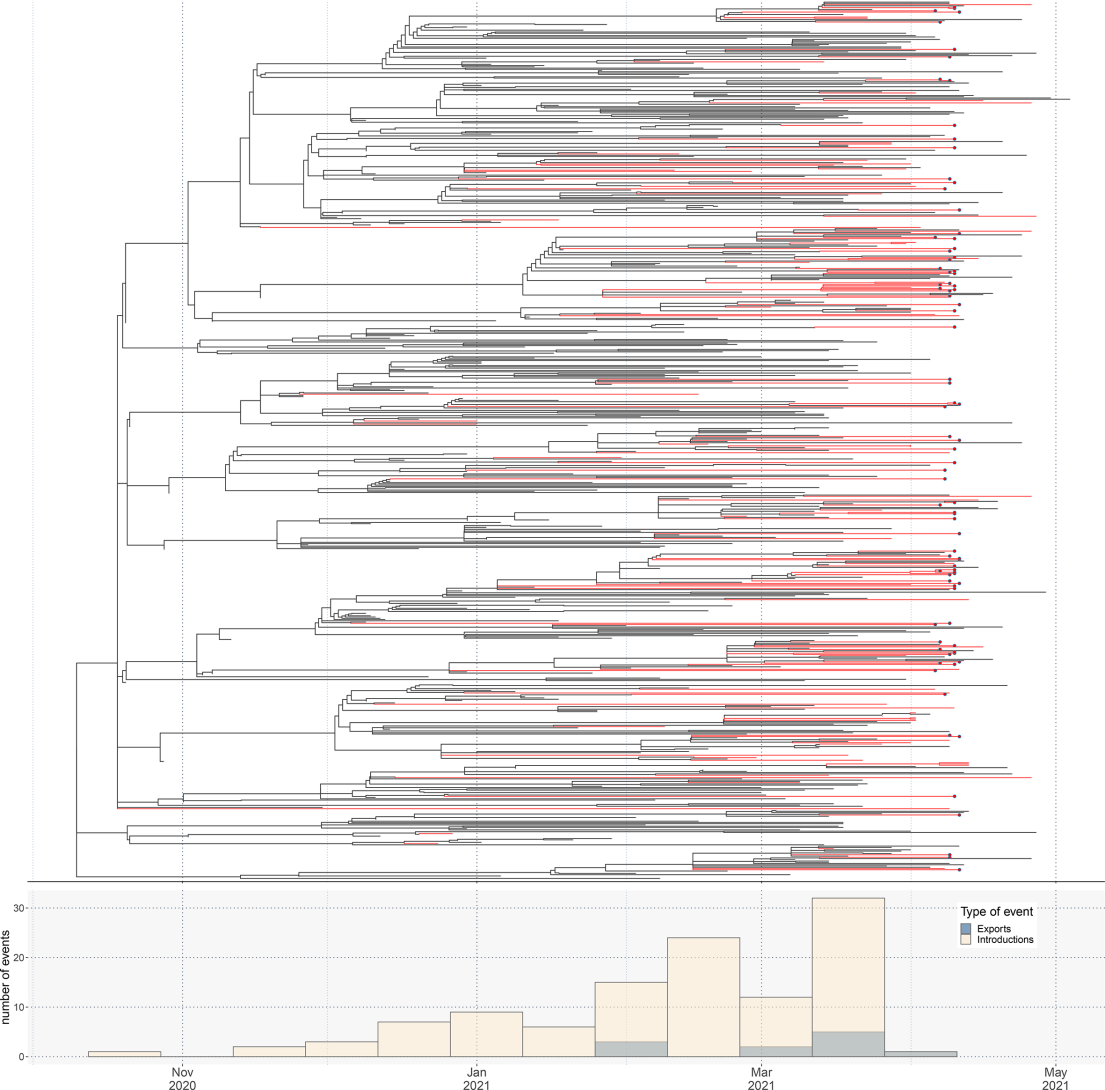
Phylogeny of B.1.1.7 (Alpha) including genomes from Pakistan. At the top, the maximum-likelihood dated tree of lineage B.1.1.7 (Alpha), showing only samples close to sequences related to Pakistan. Branches are coloured red when all descendant tips have Pakistan as the country of exposure. Samples sequenced in the present study are highlighted as red dots. At the bottom we have the histogram of introductions into and exportations out of Pakistan over time, as estimated by ancestral state reconstruction. Phylogenetic tree was estimated with IQTREE2 followed by divergence times estimation using TreeTime after excluding outliers. The figure was plotted with ggtree and introduction events were estimated by ancestral reconstruction under parsimony with the R package castor.

By reconstructing the exposure history over the phylogenetic tree, we estimate the number of Alpha importations as 112 using the dated tree, as well as 11 exports out of the country (Fig. 1). From the histogram at the bottom of Fig. 1, we observe that the first introduction of Alpha into Pakistan may have happened as early as mid October 2020. This is after the date of emergence of Alpha in the UK, which has been calculated as September 2020 elsewhere [30]. There was an increasing number of independent introductions more recently, such that most Alpha samples can be traced back to a recent ancestor abroad, while a few may have been circulating for longer (or belong to unsampled groups).

**Fig. 2.**
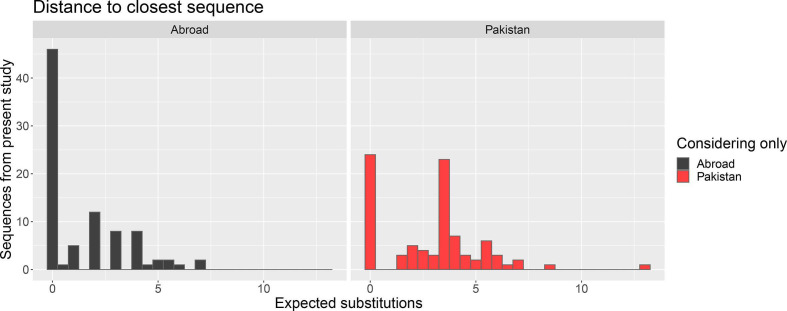
Distribution of distances to closest neighbours from Pakistan and abroad, for B.1.1.7 (Alpha) sequences. The number of expected substitutions is calculated from the patristic distances between leaves over the maximum-likelihood tree, transformed from substitutions per site by multiplying by genome length. For each of the 88 UHS-PAK sequences, we find the closest distance considering only Pakistan (blue) or international (red) tips on the tree.

Genomes from Pakistan were intermingled with genomes from elsewhere, as shown in Fig. 2 where we calculated the patristic distance of genomes in this study to their closest neighbour and found that they were generally closer to samples from abroad, i.e. samples without a travel history to Pakistan (see also Fig. S5, with the maximum-ikelihood tree). The number of substitutions to the nearest neighbour also varied (0–8 substitutions) (Fig. 2). All samples in this study were sourced from cases with no travel history. The virus is detected and sequenced from the source, usually the UK, where 2 % of the population describe their ethnicity as Pakistani [22] and where there are many daily direct flights, and then in some cases sampled in Pakistan immediately or after several months, where the number of substitutions were lower or higher, respectively.

**Fig. 3.**
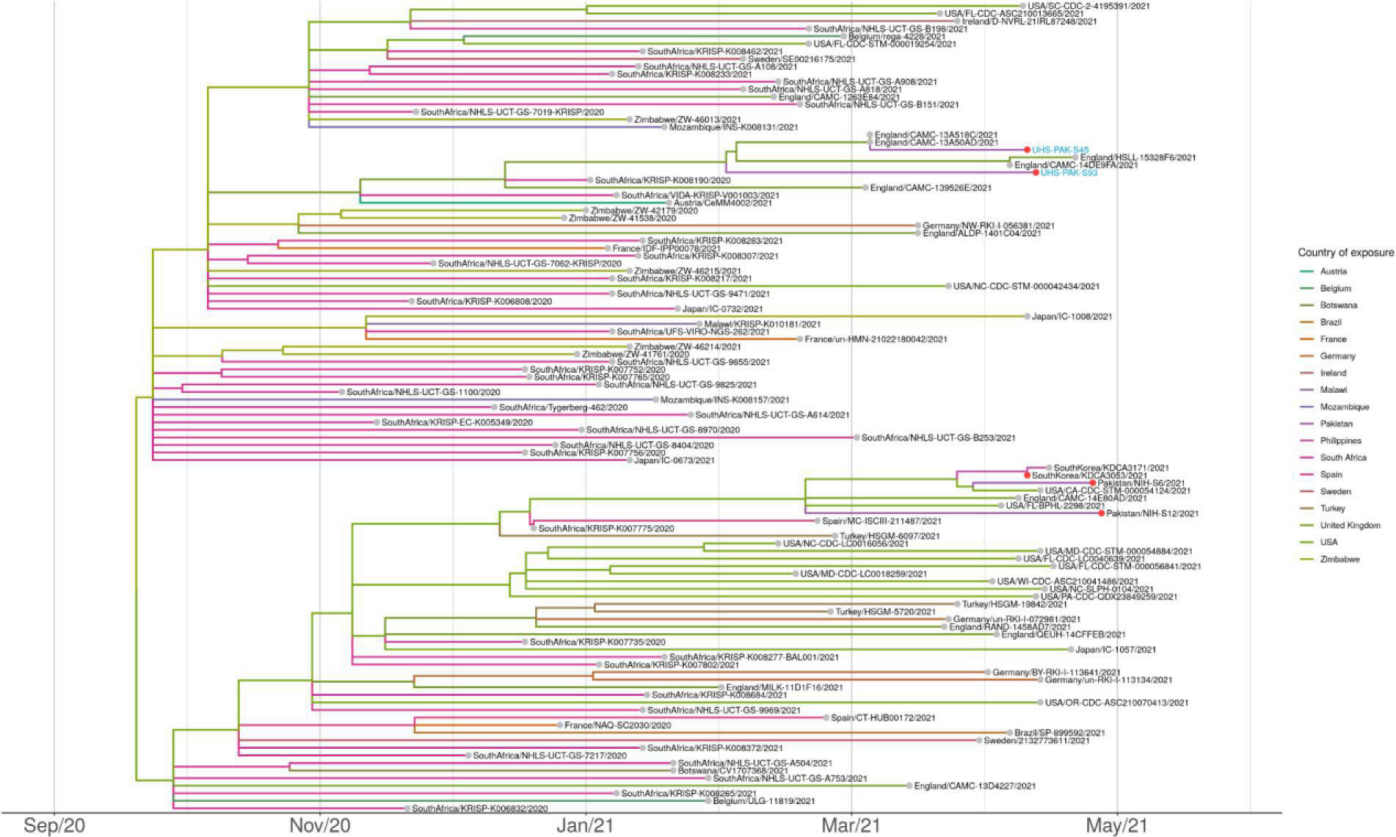
Maximum-likelihood dated tree of lineage B.1.351 (Beta) focused on samples sequenced in this study. Branches are coloured according to country of exposure, with Pakistan highlighted with a red circle. The two samples from the current study, UHS-PAK-S45 and UHS-PAK-S93, are labelled in blue. Phylogenetic tree was estimated with IQTREE2 [23] followed by divergence times estimation using TreeTime after excluding outliers. The figure was plotted with ggtree [27].

Beta was likely introduced into Pakistan recently. Beta samples were found in four clades in the phylogeny of Beta, suggesting at least four separate introductions into the country (Fig. 3). The time to the most recent common ancestor of Beta globally was calculated here as during September 2020, whereas the clades containing Beta genomes from Pakistan date no earlier than February 2021 (Fig. 3). The date of emergence of Beta is before these dates and has been calculated as early August 2020 elsewhere [8].

It has been demonstrated [31] that vaccines, in particular BNT162b2, are effective against Alpha. Therefore, if this remains the dominant lineage in the region, public health vaccination policy can be implemented accordingly. However, whilst the prevalence of Beta, which has been linked to lower vaccine efficacy, is very low, this reinforces the need for prospective surveillance of SARS-CoV-2 using genome sequencing to inform public health interventions in a continual manner.

The most regular source of genomic sequencing is from travellers from Pakistan being sequenced by their destination countries, and being annotated as such in the public databases (GISAID). Japan is the largest contributor of genomes from travellers originating from Pakistan (111 out of 269). This indirect surveillance is useful but unreliable as travel restrictions and pre-flight testing can bias the results.

This report shows the critical importance of whole-genome sequencing of SARS-CoV-2 to determine the prevalence and changing epidemiology of different variants of the virus. This data is crucial to inform public health decision makers as well as to allow global epidemiology to be understood. Building capacity for sequencing and analysis of genomes in countries with high infection rates will be crucial for the global response to COVID-19.

## Supplementary Data

Supplementary material 1

Supplementary material 2

Supplementary material 3

Supplementary material 4

Supplementary material 5
