# Supplementary material for: SARS-CoV-2 variants of concern dominate in Lahore, Pakistan in April 2021
Source: Microb Genom. 2021 Nov 30;7(11):000693. doi: 10.1099/mgen.0.000693 (PMC8743565; doi:10.1099/mgen.0.000693)

Consensus tree for maximum likelihood analysis, with ultrafast bootstrap support values and polytomies for low support nodes

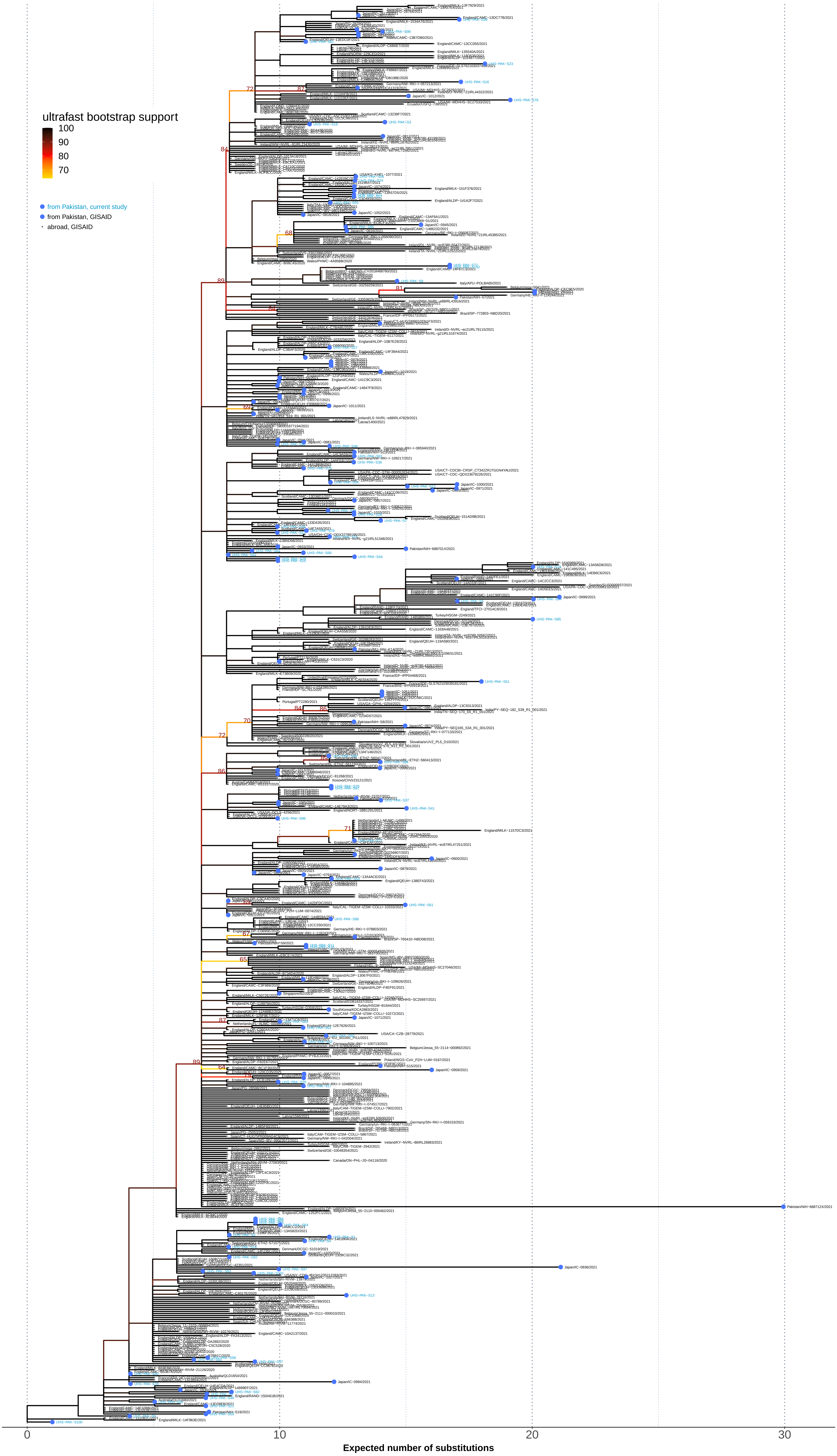

Supplement: Supplementary material 1 [file mgen-7-0693-s001.pdf]
